# Attitudes and Beliefs Toward Computerized Cognitive Training in the General Population

**DOI:** 10.3389/fpsyg.2020.00503

**Published:** 2020-04-03

**Authors:** Vina M. Goghari, Daniel Krzyzanowski, Sharon Yoon, Yanni Dai, Deanna Toews

**Affiliations:** ^1^Department of Psychology, University of Toronto Scarborough, Toronto, ON, Canada; ^2^Graduate Department of Psychological Clinical Science, University of Toronto, Toronto, ON, Canada

**Keywords:** cognitive training, public opinion, attitudes, expectations, motivation, psychological factors

## Abstract

**Introduction:**

In recent years, computerized cognitive training (CCT) programs have been developed commercially for widespread public consumption. Despite early enthusiasm, whether these programs enhance cognitive abilities in healthy adults is a contentious area of investigation. Given the mixed findings in the literature, researchers are beginning to investigate how beliefs and attitudes toward CCT impact motivation, expectations, and gains after cognitive training.

**Method:**

We collected survey data from 497 North American participants from Amazon’s Mechanical Turk (MTurk). This survey asked novel questions regarding respondents’ beliefs about the effectiveness of CCT for improving different domains of cognition, mood, and daily life; beliefs about whether CCT programs are supported by research; and whether impressions of CCT have improved or worsened over time. Exploratory analyses are reported descriptively, while parametric tests were used to analyze *a priori* hypotheses.

**Results:**

Almost half of the surveyed participants had used CCT, and respondents with a self-reported psychological or neurological disorder were more likely to have used CCT platforms than participants without such conditions. Motivations for using CCT included curiosity; to improve or maintain cognition; to prevent cognitive decline; and/or for enjoyment or fun. Participants believed that CCT is *somewhat* effective for improving mood and cognition across a variety of domains. Greater age and fewer years of education predicted perceived effectiveness of CCT. Finally, participants largely reported unchanged opinions of CCT platforms over time.

**Conclusion:**

Our study suggests the need for future research regarding the general population’s beliefs and attitudes toward CCT, along with knowledge translation for relevant stakeholders.

## Introduction

Cognitive training is a billion-dollar industry with many prominent online training platforms, including Lumosity (Lumos Labs, 2007), Peak (Brainbow Limited, 2014), Elevate (Elevate Inc, 2014), and CogniFit Brain Fitness (Cognifit, 1999). Healthy cognition is consistently associated with academic, social, and vocational success (Gottfredson, 1997). Computerized cognitive training (CCT) involves completing structured tasks that are intended to maintain or enhance specific cognitive abilities (e.g., attention, working memory), as well as fluid intelligence (i.e., the ability to reason and think logically) (von Bastian and Oberauer, 2014; Melby-Lervåg et al., 2016). Public interest in CCT has grown rapidly, since the potential to enhance cognitive abilities has widespread appeal across various populations, from individuals with typical cognitive abilities to those experiencing cognitive dysfunction or decline.

Attesting to the popularity of CCT products, Lumosity’s website claims that 100 million individuals have used its platform over a 10-year period (Lumos Labs, 2019). However, despite early positive reports and meta-analyses regarding the effects of brain training on cognitive abilities (e.g., Karbach and Verhaeghen, 2014; Au et al., 2015), the majority of meta-analyses, including the largest and most recent, have yielded null results (e.g., Melby-Lervåg and Hulme, 2013, 2016; Melby-Lervåg et al., 2016; Sala and Gobet, 2019). Additionally, the positive findings of some studies are disputed, with critics calling for greater rigor in research designs (e.g., using active control groups, as well as multiple cognitive tests to measure each construct) and in analytic techniques (e.g., controlling for multiple comparisons; accurately interpreting interactions; Lawlor-Savage and Goghari, 2014; Redick, 2015).

With many of their claims falling into disrepute among the scientific community, developers of some of the most popular CCT applications have found themselves at the center of legal controversy as well. For instance, in 2016, the Federal Trade Commission arrived at a two-million-dollar settlement with the developers of Lumosity (Lumos Labs) for misleading the public by suggesting that their application would improve users’ school/work performance and reduce or delay age-related cognitive impairment (Federal Trade Commission, 2016). Moreover, popular, credible and widely disseminated media sources have reported both this story specifically (e.g., Etchells, 2016; Entis, 2017; Gallegos, 2017) and the contentious nature of claims made by CCT developers more generally (e.g., Weeks, 2014; Zaleski, 2018; Frakt, 2019). Despite the increasing controversy surrounding CCT applications, they are still widely used among the general population, suggesting that many still believe in the utility and effectiveness of CCT for enhancing or improving cognition.

In light of these developments, researchers have begun to focus on psychosocial factors that might explain the conflicting findings regarding the efficacy of CCT interventions. A small but growing literature on psychosocial factors that influence cognitive training outcomes has concentrated mainly on several overlapping areas. Selected scholars have focused on individuals’ subjective perceptions of change after cognitive training and have generally observed self-reported improvement (Preiss et al., 2010; Stepankova et al., 2012; Goghari and Lawlor-Savage, 2018). Researchers have found that expectations regarding the impact of cognitive training–that is, the placebo effects of cognitive interventions—may influence effort and persistence such that individuals who have engaged in training may believe that they have improved and subsequently work harder during post-testing to confirm their belief (Shipstead et al., 2012; Boot et al., 2013). Other studies have found that higher levels of intrinsic motivation are associated with greater attraction to cognitive training programs, as well as higher levels of effort and persistence during use (Schweizer et al., 2011; Zhao et al., 2011; Burgers et al., 2015). A large body of research has also suggested that the need for cognition (i.e., intrinsic enjoyment of effortful cognitive activities; Cacioppo and Petty, 1982; Cacioppo et al., 1984) is positively associated with more effortful engagement in cognitive tasks (Cacioppo et al., 1996). Finally, implicit beliefs about intelligence as either a fixed/innate trait (entity theory) or a malleable trait (incremental theory; Hong et al., 1999) are also associated with CCT outcomes; individuals with fixed theories of intelligence tend to direct less effort toward tasks that are cognitively taxing as compared to incremental theorists (Grant and Dweck, 2003; Blackwell et al., 2007). Notably, both the need for cognition and implicit beliefs about the plasticity of intelligence are positively associated with participation in cognitive training protocols and training-related gains (Jaeggi et al., 2014; Foroughi et al., 2016). In summary, psychosocial factors are critical to understanding both the process and outcomes of cognitive training.

In addition, since individuals’ expectations are important predictors of playing and persisting in using the games included in cognitive training programs (Finniss et al., 2010), there is research value in characterizing their attitudes and beliefs regarding cognitive training. Moreover, a better understanding of the beliefs and attitudes of individuals who use cognitive training may provide insight regarding who is most likely to perceive benefit from these online platforms. A limited number of studies have focused on this question. A study examining adults’ optimism about brain training found that 69% of individuals believed cognitive training would be “somewhat” to “completely” successful in improving general cognition, with older adults expressing relatively greater optimism (Rabipour and Davidson, 2015). A second study of more than 3,000 younger adults who owned smartphones demonstrated that 56% had used a brain-training application (app). Of those, 65–69% reported that brain training apps improved their thinking, attention and memory, while 53% reported that the apps had a positive effect on their mood (Torous et al., 2016). This study also found that both app-naïve and app-exposed participants endorsed similarly positive beliefs regarding the effects of using these apps.

The current study’s objectives were to address the following questions regarding CCT: (1) the percentage and demographic characteristics of members of the general population who use CCT; (2) among those who have used CCT, their motivations for initiating and continuing its use; (3) whether participants’ perceptions regarding the efficacy of CCT differ depending on whether they have previously used online training platforms; (4) whether demographic characteristics (e.g., age, sex, education level) predict participants’ usage of CCT programs and their perceptions regarding the utility of such programs; and (5) whether participants’ beliefs regarding the positive effects of CCT have changed over time. The first two objectives were descriptive. For objective 3, based on previous research suggesting that a large proportion of participants believe CCT programs improve cognition and mood (e.g., Torous et al., 2016), we hypothesized that participants would generally believe that CCT is helpful. We also explored whether short-term users, long-term users, and never users of CCT would differentially rate the utility of the online programs.

For objective 4, given past findings suggesting that age positively predicts optimism regarding CCT outcomes (Rabipour and Davidson, 2015; Boot et al., 2016), we hypothesized that older age would predict more favorable beliefs about CCT effectiveness. We also added exploratory demographic predictors to this analysis to assess the predictive value of participant sex, annual income, and years of education to beliefs about CCT effectiveness to forward the literature in this area. For objective 5, because credible and widely disseminated news sources continue to report on the lack of empirical support for CCT to produce claimed benefits (Etchells, 2016; Entis, 2017; Gallegos, 2017), and because these media sources are known to impact public opinion (e.g., Jensen, 2008; Takahashi and Tandoc, 2016), we hypothesized that participants would describe their opinion of the effectiveness of CCT as having decreased over time.

## Materials and Methods

### Participants

A convenience sample of 534 participants from North America was recruited from Amazon’s Mechanical Turk (MTurk) website. This popular online crowdsourcing platform enables researchers to collect large amounts of self-report data and is commonly used across a variety of disciplines, including clinical and social sciences (Crump et al., 2013; Chandler and Shapiro, 2016). Samples were stratified by region (Canada and America) and age (18–25, 26–30, 31–35, 36–45, 46–55, and age 56 or older), with the goal of ensuring representation from each region and age group.

### Measures

#### Beliefs and Attitudes Toward CCT Questionnaire

Building on instruments used in previous research by Torous et al. (2016), we designed a 45-item self-report survey specifically for this study (see Supplementary File for full survey). We chose to build on and extend this instrument to facilitate replication and to ask novel questions regarding more specific attitudes and beliefs toward CCT. Participants responded to questions associated with the following categories:

##### Demographics

Twelve items queried demographic characteristics using open-ended and multiple-choice questions (e.g., age, income, sex, and education).

##### Prior knowledge of CCT

One item queried where participants had heard of CCT, if at all, using a multiple-choice list of 15 predetermined options (e.g., friends, family, popular media, scientific journals), including an *other* (*please specify*) option for sources not contained in the questionnaire. Also, respondents rated their level of knowledge and understanding of CCT using a seven-point scale, with endpoints labeled *1-no knowledge and understanding* and *7-excellent knowledge and understanding*.

##### History of CCT use

Six items queried history of CCT use, if any. First, participants indicated if they were *currently using*, had *previously used*, or had *never used* CCT programs using a multiple-choice format. Current and past users then indicated their duration and frequency of use by selecting one of eight multiple-choice options, ranging from *less than 1 week* to *more than 1 year* for duration and *at least once a day* to *less than once per month* for frequency. Two items queried reasons for commencing and continuing CCT use from a list of eight response options (e.g., *to maintain my level of cognitive ability, to enhance my level of cognitive ability, Curiosity in the cognitive training program/app*). An *other* (*please specify*) option was available to participants who commenced or continued use for reasons not listed in the questionnaire. Finally, participants who had never used CCT were asked if they would consider using CCT in the future and, if yes, to indicate hypothetical reasons for using CCT applications by selecting one or more of the same options presented to past and present users.

##### Knowledge and use of specific CCT applications

Two items queried which CCT programs participants had heard of and/or used in the past. The first item asked participants to select the applications that they had heard of from a list of 17 response options (e.g., Lumosity, Elevate, and CogniFit). A *none of the above* option was available to participants with no prior knowledge of such applications, and an *other* (*please specify*) option was available to those who had heard of CCT applications not listed in the questionnaire. Using the same list of options, participants were then asked which CCT applications they had used in the past, if any.

##### Psychiatric and/or neurological history

One item asked participants whether they had a psychiatric and/or neurological history that had compromised their cognition, using the following response options: (*a*) *No, I never had any;* (*b*) *Yes, I currently have;* (*c*) *Yes, I previously had;* (*d*) *Yes, but it did/does not affect cognition; and* (*e*) *If one of “Yes” options above selected, please specify.*

##### Satisfaction with CCT among past and present users

Two items asked current and past users to rate their satisfaction with CCT and whether the applications delivered expected cognitive benefits. The first item regarding satisfaction was rated on a seven-point scale, from *1-very dissatisfied* to *7-very satisfied*, with a *not applicable* option available to never users of CCT. The second item asked whether the applications delivered expected cognitive benefits, rated on a seven-point scale, with scale points labeled 1-*greatly less than expected, 4-matched expectations*, and *7-greatly exceeded expectations*, again with a *not applicable* option available to never users of CCT.

##### Beliefs in CCT effectiveness

Eleven items probed beliefs of CCT effectiveness for improving overall and domain-specific cognition, mood and everyday functioning. The domains queried included overall cognitive functioning, multi-tasking, attention, reasoning, memory, social cognition, near and far transfer, intelligence, mood, and day-to-day functioning. For each item, participants were given a brief definition (e.g., *Memory can be broadly described as the ability to use past information/knowledge in the service of the present.*) before being asked to rate CCT’s effectiveness for improving cognition within the domain. All 11 items were rated on seven-point scales, with scale points labeled *1-no*, *4-somewhat*, and *7-immensely.*

##### Beliefs of pre-existing attitudes and motivation for CCT outcomes

Two items asked participants whether prior positive beliefs about CCT, and motivation to complete CCT, impact perceived gains following use of the online programs. Each item was rated on a seven-point scale, with scale points labeled *1-no*, *4-somewhat*, and *7-immensely*.

##### Negative side effects, research support, changing opinions, and concerns of CCT use

Using a seven-point scale with endpoints labeled *1-no* and *7-immensely*, four items queried whether participants believed that CCT has negative or harmful side effects, whether CCT changes the brain, whether claims of CCT effectiveness are supported by research, and whether opinions of CCT have changed for the worse over time. Also, participants were asked to select potential concerns associated with CCT use from a multiple-choice list (e.g., cost, uncertainty regarding effectiveness, insecurity of health data). An *other* (*please specify*) option was available for participants to voice concerns not listed in the questionnaire, and a *none of the above* option was available to those who had no concerns associated with CCT use.

##### Response validity

Two response validity items served as checks against careless or non-diligent response patterns (for rationale, see Meade and Craig, 2012). The first (item 32 of 45) asked participants to respond with *“4 – somewhat”* on a 7-point Likert scale, with the selection of an unprompted response suggesting a random or haphazard response style. The second response validity item was administered at the end of the survey (item 44 of 45) and read as follows: *Last, it is vital to our study that we only include responses from people that devoted attention and effort to this study. Your answer on this question will not affect your compensation. In your honest opinion, should we use your data? Yes or No.* Participants selecting an unprompted response on item 32, or answering *No* to item 44, were removed from subsequent analyses.

### Procedure

This study was approved by the University of Toronto Research Ethics Board. North American workers matching stratified inclusion criteria on MTurk were sent an invitation to participate in the study via a secure weblink to a survey constructed using SurveyMonkey (SurveyMonkey, 2019). Following online consent, participants were free to exit the survey or to skip any item without penalty. All 45 items were delivered to all participants in the same order. After submitting the survey, participants were debriefed and were provided the principal investigator’s contact information in case of questions or concerns. Finally, participants were redirected to the MTurk batch page, where they entered their MTurk IDs one last time to verify task completion, which we retained as proof of compensation ($1.00 U.S.). The MTurk ID allows for de-identification of survey data, facilitates electronic compensation, and safeguards against the same participants taking the survey multiple times. Data were collected between October 29, 2018 and January 31, 2019.

### Statistical Analyses

Data collected via SurveyMonkey were analyzed with the Statistical Package for the Social Sciences (SPSS) Version 25 (IBM Corp, 2017). Participants failing either of the two validity response items described above, as well as participants who consented to the study but did not subsequently complete any survey items, were excluded from final analyses. A missing value analysis demonstrated missingness of less than or equal to two percent for variables that were applicable to all participants (i.e., both users and non-users of CCT). Little’s test for data missing completely at random (MCAR) suggested that data with regard to continuous variables were missing completely at random, *X*^2^(661) = 675.64, *p* = 0.34; hence, no subsequent adjustments were performed.

Demographic differences between included and excluded participants, as well as hypothesis tests of the two comparison groups, were compared via *t-*tests and chi-square tests where appropriate. One-way analyses of variance (ANOVA) were used for comparisons of three or more groups. Levene’s test addressed equal-variance assumptions, while normal sampling distributions were presumed based on a moderately large sample size (i.e., central limit theorem). Classical Cohen’s *d* calculations were computed for between-group differences where applicable.

A mixed-model ANOVA was used to address between- and within-group differences in beliefs regarding the effectiveness CCT for improving domain-specific cognitive functioning. Although statistical assumptions of normality and homogeneity of variance were met, the assumption of sphericity was violated as indicated by Mauchly’s test of sphericity, *X*^2^(35) = 143.12, *p* < 0.001, ε = 0.91. Model statistics were therefore analyzed with the Huynh-Feldt correction, as suggested by Girden (1992) when epsilon values exceed.75. Multiple comparisons were conducted with Bonferroni corrections. Within-group effect sizes and their associated standard errors were computed based on Morris and DeShon (2002) method.

A multiple linear regression analysis was used to examine demographic predictors of beliefs about CCT effectiveness. Because our only formal hypothesis concerned age as a predictor of perceived overall CCT effectiveness, the analysis was conducted in a hierarchical fashion, with age entered in Model 1, and age plus the remaining predictors entered in Model 2. All predictors were continuous except for sex, which was coded dichotomously as 1 (male) versus 2 (female).

## Results

### Participants

Of the 534 respondents, 24 were removed due to missing data (i.e., provided consent before immediately exiting the survey), and 13 participants failed one or both validity-check items. Four hundred ninety-seven participants were therefore included in final analyses (see Table 1). Chi-square and independent-samples *t*-tests showed no significant differences between included and excluded responders on any of the demographic variables assessed. Mean survey response time was 8.43 (*SD* = 5.82) min.

**TABLE 1:** Descriptive Statistics and Cognitive Training History of Participants.

	Canada	United States	Total
*N*	103	394	497
Percent Female	51.5	53.7	53.1
Age *M* (*SD*)	37.11 (11.49)	41.05 (14.29)	40.18 (13.75)
Years Education *M* (*SD*)	15.98 (3.08)	15.19 (2.20)	15.36 (2.43)
Annual Income *Mdn Range*	$38,000−$46,000	$40,000−$50,000	$40,000−$50,000
Past Users of Cognitive Training Percent	50.5	33	36.6
Current Users of Cognitive Training Percent	2.9	4.3	4.0
Duration of Use Among Users *Mdn Range*	7−30 days	7−30 days	7−30 days
Frequency of Use Among Users *Mdn*	≥2 uses per week	≥2uses per week	≥2 uses per week

*Canadian income adjusted to average American exchange rate at time of data collection. M, mean; SD, standard deviation, Mdn, median.*

### Objective 1: Frequency of CCT Use and User Demographics

Table 1 presents demographic information, proportions of past, current and total users of CCT, and average duration/frequency among users by country (Canada and United States). Briefly, participants living in Canada were significantly younger, *t*(493) = −2.42, *p* = 0.016, slightly more educated, *t*(485) = 2.93, *p* < 0.01, and more likely to have used CCT programs in the past compared to those living in the United States, *X*^2^(1, *N* = 497) = 10.76, *p* < 0.01. No significant differences in sex or current use of cognitive training were observed between Canadian- and American-dwelling participants. In total, 40.6% of the surveyed MTurk workers had used, or were currently using, CCT programs. The majority of respondents had spent one month or less using CCT (55.2%), with an observed range from less than 1 week (27.9%) to greater than 1 year (9.5%). In terms of frequency, the majority of participants (65.6%) engaged with CCT two or more times per week, with an observed range of less than once per month (14.1%) to at least once per day (29.2%).

**FIGURE 1:**
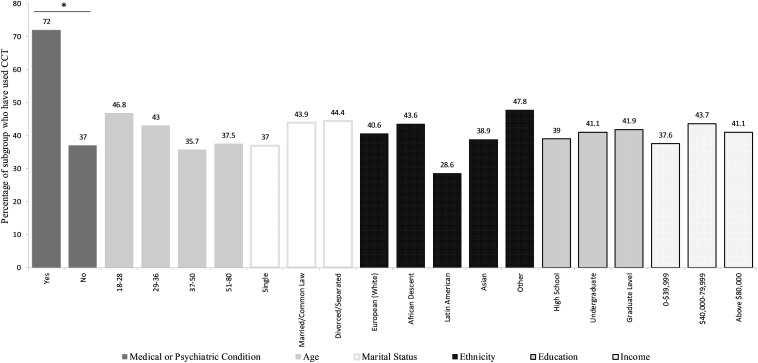
Relative frequencies of characteristics of computerized cognitive training (CCT) users by demographic category. *p < 0.01.

Figure 1 summarizes the characteristics of participants with a history of using cognitive training programs. Of the 497 included participants, 50 (10%) reported having a previous or current psychological or neurological disorder reported to cause cognitive deficits. Furthermore, these respondents were significantly more likely to have used cognitive training (72%) compared to participants who did not report having a mental or neurological disorder (37%), *X*^2^(1, *N* = 496) = 10.76, *p* < 0.01. There were no statistically significant differences between users and non-users regarding age, marital status, ethnicity, level of education, or income.

### Objective 2: Motivations for Using CCT

Table 2 summarizes the motivations for initiating use among current, past, and never users of CCT, as well as reasons for continuing use among past/present users. The most commonly endorsed reasons to engage in CCT among both users and never users, from highest to lowest frequency, were curiosity; to enhance cognition; to prevent cognitive decline; to maintain cognitive abilities; to restore or rehabilitate perceived losses in cognitive abilities; and/or for enjoyment or fun. The most commonly endorsed reasons for continuing to use CCT among current and past users, from highest to lowest frequency, were for enjoyment or fun; to enhance cognition; to prevent cognitive decline; to maintain cognitive abilities; and/or to restore or rehabilitate perceived losses in cognitive abilities.

**TABLE 2:** Relative Frequency Distributions of Reasons for Commencing and Continuing Use of Computerized Cognitive Training (CCT) Programs among Never Users and Past or Present Users.

Reason for Use	Reasons to Start CCT among Never Users Percent	Reasons to Start CCT among Past/Present Users Percent	Reasons for Continuation of CCT among Past/Present Users Percent
Curiosity	43.7	58.4	–
Enhance Cognition	42.4	49.0	40.1
Prevent Cognitive Decline	34.9	29.7	27.7
Maintain Cognition	27.8	23.3	26.7
Restore Cognitive Abilities	8.5	8.9	9.9
Enjoyment	1.0	2.0	57.9
Other	1.0	2.0	5.4

### Objective 3: Perceived Effectiveness of CCT

On average, participants believed that the effectiveness of CCT is *somewhat supported* by research (*M* = 3.83, *SD* = 1.55), as well as *somewhat effective* for improving day-to-day activities or duties (*M* = 4.00, *SD* = 1.64), mood (*M* = 3.96, *SD* = 1.55), and for enhancing areas of cognition not directly trained (i.e., far transfer; *M* = 3.81, *SD* = 1.66). There were no significant differences between past/present users and never users in their beliefs regarding CCT effectiveness in any of these domains.

To assess perceived effectiveness of domain-specific cognition, we performed a 2 × 6 mixed-model ANOVA to test for any interaction between user group (current/past users vs. never users) and perceived effectiveness of CCT for specific cognitive domains (attention, memory, reasoning, multi-tasking, intelligence, and social cognition). The purpose of this analysis was to determine (a) whether participants’ use history was associated with more positive beliefs regarding CCT effectiveness for improving different domains of cognition, and (b) whether participants believed that some cognitive domains are more amenable to positive effects of CCT than others (see Table 3 for descriptive statistics and multiple comparisons).

**TABLE 3:** Results Of 2 × 6 (User Group × Perceived Effectiveness) Mixed-Model ANOVA On Participants’ Beliefs Regarding Effectiveness of Computerized Cognitive Training (CCT) For Improving Domain-Specific Cognition.

Cognitive Domain	CCT Never Users *M* (*SE*) *N* = 292	CCT Past/Present Users *M* (*SE*) *N* = 193	Regardless of Use History *M* (*SE*) *N* = 485	Within-Subject Pairwise Comparisons Regardless of Use History Standard Mean Difference *d* (*SE*)				
				1	2	3	4	5
(1) Attention	4.22 (0.09)	4.46 (0.11)	4.34 (0.07)	–	–	–	–	–
(2) Memory	4.22 (0.09)	4.46 (0.12)	4.34 (0.08)	0 (0.06)	–	–	–	–
(3) Reasoning	3.97 (0.09)	4.09 (0.11)	4.03 (0.07)	0.27 (0.06)*	0.25 (0.06)*	–	–	–
(4) Multi-tasking	3.92 (0.09)	3.95 (0.11)	3.94 (0.07)	0.35 (0.06)*	0.29 (0.06)*	0.08 (0.06)	–	–
(5) Intelligence	3.68 (0.10)	3.86 (0.13)	3.77 (0.08)	0.42 (0.07)*	0.41 (0.06)*	0.21 (0.06)*	0.11 (0.06)	–
(6) Social cognition	3.49 (0.10)	3.36 (0.12)	3.42 (0.08)	0.68 (0.07)*	0.61 (0.07)*	0.52 (0.07)*	0.38 (0.06)*	0.24 (0.06)*

M, mean; SE, standard error. * p < 0.001. Bold items represent statistically significant pairwise differences.

Across the entire sample and across all six cognitive domains, on average, participants rated CCT as *somewhat effective* (*M* = 3.96, *SD* = 1.38) for improving or enhancing cognitive abilities. There was no statistically significant between-subject main effect of user group on perceived effectiveness of CCT across domains, *F*(1,483) = 0.82, *p* = 0.37, suggesting that past/present users and non-users of CCT share similar beliefs regarding domain-specific CCT effectiveness. However, a significant within-subject main effect was found for perceived effectiveness of CCT, *F*(5,479) = 54.789, p < 0.001. *Post hoc* multiple comparisons with Bonferroni corrections revealed that, regardless of use history, participants believed that attention and memory are most amenable to positive change through CCT use, followed by reasoning, multi-tasking, intelligence, and social cognition. Finally, the user group × perceived effectiveness of CCT interaction was significant, *F*(5,479) = 2.267, *p* = 0.047. Visual inspection of the pattern of means revealed that, although the rank order of effectiveness beliefs across cognitive domains were the same for both user groups, past/present users ranked CCT as less effective for improving social cognition compared to never users, while never users ranked CCT as less effective for improving attention, memory, reasoning, multi-tasking, and intelligence compared to past/present users.

To explore whether individuals who had used CCT for short periods and discontinued would rate it lower in usefulness than longer-term users or never users of CCT, we split our sample into three groups based on duration of use (never used, *n* = 292; used for less than 1 month, *n* = 110; and used for greater than 1 month, *n* = 91). We then examined average ratings of overall perceived effectiveness of CCT for improving cognition, using the following item rated on a 7-point Likert scale: *Regardless of use, do you think cognitive training programs enhance overall cognitive function or abilities?* Participants who had never used the online programs (*M* = 4.07, *SD* = 1.39) and those who had used CCT for less than 1 month (*M* = 3.97, *SD* = 1.34) gave similar ratings of overall CCT effectiveness (*d* = 0.07). However, participants who had used CCT for more than 1 month (*M* = 4.52; *SD* = 1.48) rated overall effectiveness of CCT considerably higher compared to never users (*d* = 0.32) and brief users (*d* = 0.40).

We also examined whether participants who had used CCT for more than 1 month were more satisfied with CCT compared to brief users. The following item, rated on a 7-point Likert scale from *very dissatisfied* to *very satisfied*, was used to address this question: *How would you rate your overall experience with cognitive training programs/apps?* Participants who had used CCT for longer periods were more satisfied (*M* = 5.35, *SD* = 1.40) compared to brief users (*M* = 4.58, *SD* = 1.35), *t*(199) = 3.96, *p* < 0.001, *d* = 0.56. Finally, using a one-way ANOVA, we tested the relationship between use history and beliefs regarding whether CCT causes negative or harmful side effects. The following item, rated on a 7-point Likert scale from *no* to *immensely*, was entered as the dependent variable: *Regardless of use, do you think cognitive training programs/apps can have negative side effects or lead to harmful effects?* The model was not significant, *F*(2,489) = 0.46, *p* = 0.63. Regardless of use history, 48.7% of participants responded *no* to this item, an additional 34.2% responded between *no* and *somewhat harmful* (non-inclusive), and 16.6% reported believing that CCT causes between *somewhat* and *immensely* harmful side effects (inclusive).

### Objective 4: Participant Characteristics and Perceived CCT Effectiveness

We performed a hierarchical linear regression analysis to test the hypothesis that age positively predicts beliefs about CCT effectiveness (Model 1), as well as to explore whether sex, annual income, and years of education predict perceived overall effectiveness of CCT for improving or enhancing cognition (Model 2; see Table 4 for complete statistics). The regression equation in Model 1 was significant, with age positively predicting perceived CCT effectiveness. The regression equation in Model 2 was also significant, with greater age and lower education each predicting positive beliefs about the effectiveness of CCT for improving or enhancing overall cognitive functioning. Neither sex nor income level provided any predictive power in this analysis.

**TABLE 4:** Regression Analysis Predicting Perceived Effectiveness of Computerized Cognitive Training (CCT) for Improving or Enhancing Overall Cognition.

	Perceived CCT effectiveness				
	*b*	*SE b*	β	*t*	*p*
**Model One**										
Age	0.02	0.01	0.15	3.366	0.001
*F*(1,479) = 11.333, *p* = 0.001, *R*^2^ = 0.02, *R*^2^ *Adj.* = 0.02					
**Model Two**					
Age	0.02	0.01	0.17	3.670	<0.001
Education (years)	−0.09	0.03	−0.15	−3.247	0.001
Sex	−0.01	0.13	−0.003	−0.072	0.94
Income	0.03	0.02	0.07	1.429	0.15
*F*(4,476) = 5.776, *p* < 0.001, *R*^2^ = 0.05, *R^2^ Adj.* = 0.04					

*SE, standard error.*

### Objective 5: Beliefs About CCT Effectiveness Over Time

While excluding respondents who had never heard of CCT at the time of study participation (*n* = 101), we used the following item, rated on a 7-point Likert scale, from *not at all* to *extremely*, as the dependent variable: *Regardless of use, has your opinion of the positive effects of cognitive training changed for worse over time?* Of the 394 respondents who had heard of CCT prior to participation, 58.3% reported that their opinions of CCT had not changed at all over time, an additional 19.7% between *not at all* and *moderately* changed opinions (non-inclusive), and the remaining 21.4% reported between *moderately* and *extremely* changed opinions of CCT for the worse over time (inclusive). We also tested whether use history was associated with changing negative perceptions of CCT over time, again excluding participants who had never heard of CCT at the time of study participation. Respondents who had previously used CCT (*n* = 190) were more likely to have changed their opinion for the worse (*M* = 2.32, *SD* = 1.77) compared with participants who had never used CCT (*n* = 204; *M* = 1.97, *SD* = 1.62), *t*(392) = 2.083, *p* = 0.04. However, although users reported an increase in negative opinions over time, the effect size was small (*d* = 0.21), and the means for both groups indicated little to no average change in opinions of CCT effectiveness.

## Discussion

Computerized cognitive training is quite popular within the general population. Lumosity, perhaps the most notable and popular cognitive training platform, purports to have had 100 million, per their first quoted total users. Given the prominence of CCT, as well as the recent controversy surrounding CCT effectiveness (Federal Trade Commission, 2016), there is a need to better understand the general population’s attitudes and beliefs toward these programs. To this end, we investigated several novel questions using an Amazon MTurk convenience sample, which provided important insights regarding those who are most motivated to use CCT and who might benefit most from engaging with these training programs.

First, to get a better sense of who uses CCT and in what capacity, this study described average rates, frequencies, and duration of CCT use among MTurk workers in North America. A sizeable proportion (40.6%) of surveyed MTurk workers reported to be currently using, or having previously used, CCT programs. Our findings resemble those of a study of younger adult users of smartphone apps, which demonstrated that 56% of individuals had used a brain training app (Torous et al., 2016). The present study also reveals that individuals across many age groups have tried CCT. Of note, although most of the sample had used CCT for a brief period, approximately 45% of users had spent anywhere from 1 month to more than 1 year using the platforms, suggesting that CCT does attract a significant proportion of long-term users in the general population. Participants also reported a consistent frequency of use, with more than 65% engaging with CCT two or more times per week.

Notably, we found that 72% of participants with a self-reported psychological or neurological condition have used CCT, making them almost twice as likely to engage with the programs compared to participants with no such conditions. Given that vulnerable individuals are especially likely to use CCT, possibly to help restore or maintain cognitive functioning, it is imperative that these individuals possess accurate information regarding the efficacy of CCT in meeting their goals. Moving forward, it would be useful to study these specific individuals within the population to determine their attitudes and beliefs regarding cognitive training.

A second goal of this project was to characterize motivations for using CCT. The leading reasons to begin using CCT included curiosity, to enhance cognition, to prevent cognitive decline, and to restore cognitive abilities. These same reasons for initiating use were endorsed at similar rates among past/present users for continuing to use CCT. Given that three of the top four reasons cited for using CCT included perceived benefits to cognition, participants appear to be largely unaware of the lack of scientific support for these programs, as well as the legal issues surrounding one of CCT’s largest developers in Lumos Labs (Federal Trade Commission, 2016; Lumos Labs, 2019). It is therefore vital that consumers have realistic expectations regarding the actual benefits of CCT, since the main justification our participants mentioned for initiating use was to maintain or restore cognitive abilities. Interestingly, while only one percent of never users, and two percent of past/present users, reported entertainment as the primary motivation to begin utilizing the online platforms, enjoyment or fun was the leading motivator for *continuing* to use CCT among current and past users. This finding suggests that CCT developers wishing to attract and retain repeat customers should more accurately advertise these programs as engaging and entertaining games, rather than as applications that improve or enhance daily cognition.

A principal aim of this study was to characterize public perceptions of the effectiveness of CCT for improving or rehabilitating daily functioning and cognition. Our first hypothesis–that participants would believe cognitive training is useful–was partially supported, as participants’ average rating for CCT effectiveness corresponded to a *somewhat effective* rating for improving or enhancing mood, day-to-day activities, and cognitive efficiency in unrelated tasks, as well as in relation to overall and domain-specific cognitive functioning. Nevertheless, while users and non-users held similar beliefs regarding the effectiveness of CCT for improving or enhancing the behavioral and cognitive domains queried, there were significant within-participant differences across cognitive domains. That is, respondents believed that memory and attention are most amenable to benefits of CCT use, followed by reasoning, multi-tasking, intelligence, and social cognition. More generally, 75.4% of our MTurk participants believed that CCT is *somewhat* to *immensely effective* for improving overall cognitive function or abilities. A previous study focusing on optimism about brain training among adults found that 69% believed cognitive training would be *somewhat* to *completely* successful at improving general cognition (Rabipour and Davidson, 2015). Another study assessing young adults’ use of smartphone apps found that 65–69% felt the apps helped their thinking, attention, and memory, and 53% felt it helped their mood (Torous et al., 2016). Similar to our study, research by Torous et al. (2016) also found that user beliefs regarding CCT did not differ from those of non-users. Our study builds on these findings by evaluating a wider range of cognitive abilities and aspects of daily functioning, and showing that relatively fundamental cognitive abilities targeted by cognitive training (e.g., attention, working memory) are perceived to be more amenable to change than are higher-level cognitive abilities (e.g., social cognition, intelligence).

Furthermore, most respondents in our sample did not believe that CCT causes negative or harmful side effects, although a small subset (approximately 16% of participants) reported that they believed CCT is either *somewhat* to *immensely* harmful. This proportion of respondents is comparable to the subset of participants in a study by Torous et al. (2016), who found that 14.9% of their sample believed there were dangers associated with app use (although app use is more generalized than CCT use). The consistency of these findings across studies is not trivial, given the large number of individuals who engage with CCT platforms. Future investigations should probe which aspects of CCT usage respondents perceive to be harmful.

We also found that individuals who had used CCT for short periods and discontinued rated it lower in usefulness than those who had sustained use or who had never used CCT. Perhaps not surprisingly, longer-term users were also more satisfied with CCT than brief users, suggesting that the perceived benefits of CCT use were higher in this group. This finding, based on a sample from the general population who chose to use CCT independently according to their preferred schedule, differs from our own previous findings in cognitive training trials. In our own cognitive training protocols, which entailed surveying participants’ attitudes and beliefs about CCT before and after they completed a cognitive training trial, greater participant use of CCT over time was associated with reduced post-trial expectations regarding its efficacy for improving aspects of daily life (Goghari and Lawlor-Savage, 2018).

We delineated which participant characteristics predict perceived CCT effectiveness for improving cognition. This question is critical to understanding who might be more likely to use and benefit from CCT. While we formally hypothesized that greater age would predict more positive attitudes, we also explored the predictive value of years of education, sex, and annual income in our model. Greater age and fewer years of education predicted more positive perceptions of CCT effectiveness, while sex and annual income provided no explanatory power in our analyses. Similar to our study, Rabipour and Davidson (2015) found that older adults perceived cognitive training as more beneficial compared to younger adults, even in the face of pessimistic information about cognitive training. It should be noted, however, that the regression model in our study explained less than five percent of variance in beliefs, suggesting that many factors not assessed using our instrument play a role in participants’ beliefs about CCT effectiveness. In addition to demographic variables, future researchers may wish to examine other individual factors that influence beliefs, initiation, and continuation of CCT use as a recreational or health-promoting behavior. For instance, dispositional factors warranting such investigation include typical/preferred intellectual engagement (Goff and Ackerman, 1992), need for cognition (Cacioppo et al., 1996), five-factor personality traits (e.g., openness and conscientiousness; Ozer and Benet-Martínez, 2006), belief in intelligence as a malleable trait (Hong et al., 1999), dispositional optimism (Glaesmer et al., 2012), intensity and perseverance in pursuing long-term goals (Duckworth et al., 2007), and coping style in response to stress or challenges (Miller, 1987; Steptoe, 1989).

Lastly, we examined whether opinions of CCT effectiveness have changed over time. Since research has demonstrated little support for CCT as an effective tool for improving or enhancing cognition (e.g., Melby-Lervåg and Hulme, 2013, 2016; Melby-Lervåg et al., 2016), and given the increase in negative press concerning CCT in recent years (Weeks, 2014; Etchells, 2016; Entis, 2017; Gallegos, 2017; Zaleski, 2018; Frakt, 2019), we predicted that participants’ opinions of CCT would have changed for the worse over time. However, our respondents generally reported little to no change in opinion, while simultaneously reporting motivations for beginning and continuing to use CCT in line with the claimed benefits of using such applications (i.e., to improve or enhance cognition, to prevent cognitive decline, and/or to remediate perceived cognitive losses). Taken together, these findings may suggest that respondents are in fact unaware of the lack of empirical support for CCT. Further to this point, participants reported that they believed CCT is *somewhat* supported by research, which may indicate that they are unsure whether CCT is, or is not, supported in the scientific literature.

Limitations of this study include the use of an online convenience sample of MTurk workers whose characteristics may differ from the overall population (Huff and Tingley, 2015; Burnham et al., 2018; Walters et al., 2018). However, using MTurk enabled us to recruit from a wide range of ages in both Canada and the United States. Additionally, it would have been useful to augment the forced-choice responses in our survey with open-ended responses for certain questions, such as what aspects of CCT participants found harmful. Our participants completed this study online and in uncontrolled environments, raising questions regarding distractions, levels of concentration and, ultimately, data quality. However, we observed very little missing data across the vast majority of respondents, and we employed response validity items to identify non-diligent responders. We are therefore confident in the quality of our data and the validity of our findings. Furthermore, a strength of the current investigation included its large sample size, which permitted us to investigate beliefs and attitudes toward CCT across a broad cross-section of ages.

## Conclusion

In short, this study addressed several novel questions, including whether participants’ perceptions of the utility of CCT differed by cognitive domain and presumed functional outcome; whether perceptions of the utility of CCT have changed over time; reasons for initiating and continuing use of CCT; and who uses CCT. This study notably found that vulnerable individuals (i.e., those with a self-reported psychological or neurological disorder affecting cognition) were more likely to use cognitive training than other members of the general public. We also observed that the general public retains a “somewhat positive” perception of the utility of cognitive training (contrary to most evidence). This finding points to the vital importance of clearly and accurately communicating the scientific consensus regarding the benefits of CCT, since a sizeable proportion of the population uses CCT and endorses its efficacy for improving, maintaining, or restoring cognitive function.

## Data Availability Statement

The datasets generated for this study are available on request to the corresponding author.

## Ethics Statement

The studies involving human participants were reviewed and approved by the University of Toronto Research Ethics Board. The patients/participants provided their written informed consent to participate in this study.

## Author Contributions

VG supervised the project, designed the study and oversaw the implementation of the study and analyses, and wrote a substantial amount of the manuscript. DK analyzed the data and wrote large sections of manuscript with support from VG. SY and YD contributed to the study design and implementation of the study and provided critical feedback on the manuscript. DT helped with writing, literature review, and critical editing. All authors read and approved the submitted version.

## Conflict of Interest

The authors declare that the research was conducted in the absence of any commercial or financial relationships that could be construed as a potential conflict of interest.
