# Supplementary material for: Attitudes and Beliefs Toward Computerized Cognitive Training in the General Population
Source: Front Psychol. 2020 Apr 3;11:503. doi: 10.3389/fpsyg.2020.00503 (PMC7147517; doi:10.3389/fpsyg.2020.00503)
Supplement: Supplementary file 1 [file Data_Sheet_1.docx]

**Supplemental File**

**Beliefs and Attitudes towards Cognitive Training Questionnaire^[[1]](#footnote-1)^**

1. What is your age?
2. What is your biological sex at birth?
   1. Male
   2. Female
   3. Intersex
3. What is your current gender identity? Please select all that apply.
4. Man
5. Woman
6. Transgender
7. Other (please specify): _______
8. What is your country of birth?
   1. Canada
   2. United States
   3. United Kingdom
   4. Australia
   5. European Union Countries or other countries
      (please specify which country):_________
9. What is your current country of residence?
10. Canada
11. United States
12. United Kingdom
13. Australia
14. European Union Countries or other countries
    (please specify which country):_________
15. What is your current marital status:
    1. Single, never married
    2. Common-Law
    3. Married, never divorced
    4. Divorced and not remarried
    5. Divorced and remarried
    6. Separated
    7. Widowed
    8. Other (please specify): _____________
16. What is the ethnicity that you identify with?

| 1. Arab |
| --- |
| 1. Black (e.g., African, Haitian, Jamaican, Somali, etc.) |
| 1. East Asian (e.g., Chinese, Japanese, Korean) |
| 1. South Asian (e.g., East Indian, Pakistani, Bengali, Sri Lankan, etc.) |
| 1. Southeast Asian (e.g., Filipino, Vietnamese, Indonesia, Malaysian, Singaporean, etc.) |
| 1. West Asian (e.g., Iranian, Afghan, etc.) |
| 1. Latin American |
| 1. Indigenous/First Nations/Inuit/Métis |
| 1. European Origin/White |
| 1. Mixed ethnic heritage/Multiracial |
| 1. Individually defined (please specify):______________ |

1. What is the highest level of education you have completed? If you’re currently enrolled in school, please indicate the highest degree you have received.
   1. No formal education
   2. Less than a high school diploma
   3. High school degree or equivalent
   4. College
   5. Vocational training
   6. Associate degree
   7. Bachelor degree
   8. Master’s degree
   9. Professional degree
   10. Doctorate
   11. Other (please specify):____________
2. In years, how many years of education have you completed (starting with elementary school)? For example, completing high school in North America would be 12 years of education.
3. What is your last year total annual household income (in US dollars) before tax deductions (gross income based on tax returns)?
   1. Under $10,000 (including loss)
   2. $10,000 to $19,999
   3. $20,000 to $29,999
   4. $30,000 to $39,999
   5. $40,000 to $49,999
   6. $50,000 to $59,999
   7. $60,000 to $69,999
   8. $70,000 to $79,999
   9. $80,000 to $89,999
   10. $90,000 to $99,999
   11. $100,000 to $149,999
   12. $150,000 and over
4. What is your current occupation (excluding working as a participant on MTurk)?
   1. Management occupations
   2. Business, finance and administration occupations
   3. Natural and applied sciences and related occupations
   4. Health occupations
   5. Occupations in education, law and social, community and government services
   6. Occupations in art, culture, recreation and sport
   7. Sales and service occupations
   8. Trades, transport and equipment operators and related occupations
   9. Natural resources, agriculture and related production occupations
   10. Occupations in manufacturing and utilities
   11. Unemployed
   12. Other (please specify, e.g., student, military service): ____________
5. Have you ever had a neurological or psychological disorder that might compromise your cognitive ability? Please select all that apply.
   1. No, I never had any
   2. Yes, I currently have
   3. Yes, I previously had
   4. Yes, but it did/does not affect cognition
   5. If one of “Yes” options above selected, please specify:____________

**Cognitive training can be described as improving, rehabilitating or preserving cognitive abilities through intentional practice. In this study we are referring to the type of cognitive training you do on a computer individually (i.e., not with a group). Please use this definition to answer the questions below.**

1. Have you ever heard of the terms “cognitive training”, “cognitive exercises”, or “brain training” apart from this study? If so, where have you heard of these terms from? Please select all that apply.
   1. No
   2. Yes, from friends
   3. Yes, from family
   4. Yes, from academic professionals
   5. Yes, from health professionals
   6. Yes, from my workplace
   7. Yes, from social media (Facebook, Instagram, Twitter etc.)
   8. Yes, from radio
   9. Yes, from television
   10. Yes, from broadcast news
   11. Yes, from advertisements
   12. Yes, from newspapers
   13. Yes, from magazines
   14. Yes, from journals
   15. Yes, but from other sources (please specify):_________________
2. How would you rate your existing knowledge and understanding of cognitive training apart from this study?

| no knowledge and understanding |  |  | some knowledge and understanding |  |  | excellent knowledge and understanding |
| --- | --- | --- | --- | --- | --- | --- |
| 1 | 2 | 3 | 4 | 5 | 6 | 7 |

1. Have you ever used a program/app designed to enhance your cognitive abilities such as attention, memory and others? In this survey, we will refer to these as cognitive training programs/apps.
   1. Currently Using
   2. Previously Used
   3. Never Used
2. If you have previously or are currently using a program/app designed to enhance your cognitive abilities, what was the longest duration you have ever used the product?
   1. N/A, I have never used it
   2. Less than one week
   3. One week to a month
   4. 1-2 months
   5. 2-3 months
   6. 3-6 months
   7. 6 months to a year
   8. 1 year or more
3. If you have previously or currently are using a program/app designed to enhance your cognitive abilities, how often have or are you using the program/app?
   1. N/A, I have never used it
   2. At least once a day
   3. 2 or more times a week
   4. Once a week
   5. 2 or more times a month
   6. Once a month
   7. Less than once a month
   8. Other (please specify):_____________
4. If you have previously or are currently using a program/app designed to enhance your cognitive abilities, what was the reason for why you joined as a user? Please select all that apply.
5. N/A, I have never used it
6. To maintain my level of cognitive ability
7. To enhance my level of cognitive ability
8. To prevent decline in my cognitive abilities
9. To rehabilitate/restore my cognitive abilities
10. Curiosity in the cognitive training program/app
11. Interest in trying the brain training activities
12. Other (please specify):________________________
13. If you have previously or currently are using a program/app designed to enhance your cognitive abilities, what were the reasons for why you continued to use the cognitive training program after you started as a user? Please select all that apply.
14. N/A, I have never used it
15. To maintain my level of cognitive ability
16. To enhance my level of cognitive ability
17. To prevent decline in my cognitive abilities
18. To rehabilitate/restore my cognitive abilities
19. Enjoyment in playing the brain training activities
20. Other (please specify):_____________

If you have never used a cognitive training program/app, would you consider using one? If so, what would be your reason for joining a program/app as a user? Please select all that apply.

1. No
2. Yes, to maintain my level of cognitive ability
3. Yes, to enhance my level of cognitive ability
4. Yes, to prevent decline in my cognitive abilities
5. Yes, to rehabilitate/restore my cognitive abilities
6. Yes, curiosity in the cognitive training program/app
7. Yes, interest in trying the brain training activities
8. Yes, but for other reasons (please specify):_____________________
9. Have you ever heard of the following the following cognitive training programs/apps? Please select all that apply.
10. Elevate
11. Lumosity
12. Peak
13. NeuroNation
14. Fit Brains
15. CogniFit
16. BrainHQ
17. Brain Wars
18. Mind Games
19. Memorado
20. HighIQPro
21. Happify
22. Memory Trainer
23. CogMed
24. Happy Neuron
25. Other (please specify):________
26. None of the above
27. Have you ever used any of the following the following cognitive training programs/apps? Please select all that apply.
28. Elevate
29. Lumosity
30. Peak
31. NeuroNation
32. Fit Brains
33. CogniFit
34. BrainHQ
35. Brain Wars
36. Mind Games
37. Memorado
38. HighIQPro
39. Happify
40. Memory Trainer
41. CogMed
42. Happy Neuron
43. Other (please specify):________
44. None of the above
45. Have you ever used a cognitive training program/app to help with compromised cognitive ability related to your neurological or psychological disorder?
    1. N/A, I have never had a neurological or psychological disorder that compromises my cognitive ability
    2. Yes
    3. No
46. How would you rate your overall experience with cognitive training programs/apps? If never used, pick “not applicable.”

| very dissatisfied |  |  | neutral |  |  | very satisfied | Not applicable |
| --- | --- | --- | --- | --- | --- | --- | --- |
| 1 | 2 | 3 | 4 | 5 | 6 | 7 |  |

1. As a consumer, do you feel that the cognitive training programs/apps that you used delivered the cognitive benefits that you expected? Did it meet your expectations? If never used, pick “not applicable.”

| greatly less than expected |  |  | matched expectations |  |  | greatly exceeded expectations | Not applicable |
| --- | --- | --- | --- | --- | --- | --- | --- |
| 1 | 2 | 3 | 4 | 5 | 6 | 7 |  |

1. Regardless of use, do you think cognitive training programs/apps enhance overall cognitive function/abilities?

| no |  |  | somewhat |  |  | immensely |
| --- | --- | --- | --- | --- | --- | --- |
| 1 | 2 | 3 | 4 | 5 | 6 | 7 |

1. Regardless of use, do you think cognitive training programs/apps improve multi-tasking abilities? Multi-tasking can be described as the ability to do multiple things simultaneously.

| no |  |  | somewhat |  |  | immensely |
| --- | --- | --- | --- | --- | --- | --- |
| 1 | 2 | 3 | 4 | 5 | 6 | 7 |

1. Regardless of use, do you think cognitive training programs/apps improve attention? Attention can be described as the ability to choose and concentrate on relevant information.

| no |  |  | somewhat |  |  | immensely |
| --- | --- | --- | --- | --- | --- | --- |
| 1 | 2 | 3 | 4 | 5 | 6 | 7 |

1. Regardless of use, do you think cognitive training programs/apps improve reasoning? Reasoning can be described as the process of thinking during which an individual aware of a problem identifies, evaluates, and decides upon a solution.

| no |  |  | somewhat |  |  | immensely |
| --- | --- | --- | --- | --- | --- | --- |
| 1 | 2 | 3 | 4 | 5 | 6 | 7 |

1. Regardless of use, do you think cognitive training programs/apps enhance memory? Memory can be broadly described as the ability to use past information/knowledge in the service of the present.

| no |  |  | somewhat |  |  | immensely |
| --- | --- | --- | --- | --- | --- | --- |
| 1 | 2 | 3 | 4 | 5 | 6 | 7 |

1. Regardless of use, do you think cognitive training programs/apps enhance social cognition (i.e., the role that cognitive processes play in social interactions)?

| no |  |  | somewhat |  |  | immensely |
| --- | --- | --- | --- | --- | --- | --- |
| 1 | 2 | 3 | 4 | 5 | 6 | 7 |

1. Regardless of use, do you think cognitive training programs/apps can enhance cognitive abilities that were not practiced/trained?

| no |  |  | somewhat |  |  | immensely |
| --- | --- | --- | --- | --- | --- | --- |
| 1 | 2 | 3 | 4 | 5 | 6 | 7 |

1. Respond with a “somewhat” for this item.

| no |  |  | somewhat |  |  | immensely |
| --- | --- | --- | --- | --- | --- | --- |
| 1 | 2 | 3 | 4 | 5 | 6 | 7 |

1. Regardless of use, do you think cognitive training programs/apps can enhance intelligence? Intelligence can be described as the ability to learn from experience, solve problems, and use knowledge to adapt to new situations.

| no |  |  | somewhat |  |  | immensely |
| --- | --- | --- | --- | --- | --- | --- |
| 1 | 2 | 3 | 4 | 5 | 6 | 7 |

1. Regardless of use, do you think cognitive training programs/apps improve mood? Mood can be described as a prevailing psychological state; a feeling, state or prolonged emotion.

| no |  |  | somewhat |  |  | immensely |
| --- | --- | --- | --- | --- | --- | --- |
| 1 | 2 | 3 | 4 | 5 | 6 | 7 |

1. Regardless of use, do you think motivation to complete training affects gain on cognitive training programs/apps?

| no |  |  |  | somewhat |  |  | immensely |
| --- | --- | --- | --- | --- | --- | --- | --- |
| 1 | 2 | 3 |  | 4 | 5 | 6 | 7 |

1. Regardless of use, do you think expectations (i.e., believing or not believing) cognitive training affects gain on cognitive training programs/apps?

| no |  |  | somewhat |  |  | immensely |
| --- | --- | --- | --- | --- | --- | --- |
| 1 | 2 | 3 | 4 | 5 | 6 | 7 |

1. Regardless of use, do you think cognitive training programs/apps improve performance in day-to-day duties/activities? For example, would repetitively playing a cognitive training memory game over a substantial length of time improve your ability to remember where you last left your keys or where you parked your car?

| no |  |  | somewhat |  |  | immensely |
| --- | --- | --- | --- | --- | --- | --- |
| 1 | 2 | 3 | 4 | 5 | 6 | 7 |

1. Regardless of use, do you think cognitive training programs/apps can have negative side effects or lead to harmful effects?

| no |  |  | somewhat |  |  | immensely |
| --- | --- | --- | --- | --- | --- | --- |
| 1 | 2 | 3 | 4 | 5 | 6 | 7 |

1. Do you think research evidence supports the claim that cognitive or brain training changes the brain?

| no |  |  | somewhat |  |  | immensely |
| --- | --- | --- | --- | --- | --- | --- |
| 1 | 2 | 3 | 4 | 5 | 6 | 7 |

1. Regardless of use, has your opinion of the positive effects of cognitive training changed for the better over time?

| not at all |  |  | moderately |  |  | extremely |
| --- | --- | --- | --- | --- | --- | --- |
| 1 | 2 | 3 | 4 | 5 | 6 | 7 |

1. Regardless of use, has your opinion of the positive effects of cognitive training changed for worse over time?

| not at all |  |  | moderately |  |  | extremely |
| --- | --- | --- | --- | --- | --- | --- |
| 1 | 2 | 3 | 4 | 5 | 6 | 7 |

1. Do you have any concerns regarding the use of cognitive training programs/apps? Please select all that apply.
2. Costs
3. Dedicated time of use
4. Uncertainty of promised effectiveness
5. No interest
6. Insecurity of health data,
7. Not supported/promoted by healthcare professionals
8. Other (please specify):________________
9. None of the above
10. Last, it is vital to our study that we only include responses from people that devoted attention and effort to this study. You answer on this question will not affect your compensation. In your honest opinion, should we use your data?
11. Yes
12. No
13. Please type in your Amazon mTurk Worker ID. This can be found on your Dashboard or in the upper left corner of the new Worker website.

1. This inventory builds on and extends the inventory created by Torous, J., Staples, P., Fenstermacher, E., Dean, J., & Keshavan, M. (2016). Barriers, benefits, and beliefs of brain training smartphone apps: An internet survey of younger US consumers. *Frontiers in Human Neuroscience, 10*, 180. doi:10.3389/fnhum.2016.00180. [↑](#footnote-ref-1)
